# Optoacoustic imaging of the breast: correlation with histopathology and histopathologic biomarkers

**DOI:** 10.1007/s00330-019-06262-0

**Published:** 2019-05-27

**Authors:** Gisela L. G. Menezes, Ritse M. Mann, Carla Meeuwis, Bob Bisschops, Jeroen Veltman, Philip T. Lavin, Marc J. van de Vijver, Ruud M. Pijnappel

**Affiliations:** 1 Seno Medical Instruments, 8023 Vantage Dr. #1000, San Antonio, TX 78230 USA; 2 Department of Radiology, Radboud University Medical Center, Geert Grooteplein Zuid 10, 6525 GA Nijmegen, The Netherlands; 3 Department of Radiology, Rijnstate Hospital, Wagnerlaan 55, 6815 AD Arnhem, The Netherlands; 4 Department of Radiology, Albert Schweitzer Hospital, Albert Schweitzerplaats 25, 3318 AT Dordrecht, The Netherlands; 5 Department of Radiology, ZGT, Zilvermeeuw 1, 7609 PP Almelo, The Netherlands; 6 Boston Biostatistics Research Foundation, 3 Cahill Park Drive, Framingham, MA 01702 USA; 7 Department of Pathology, Netherlands Cancer Institute, Plesmanlaan 121, 1066 CX Amsterdam, The Netherlands; 8 Department of Radiology and Nuclear Medicine, University Medical Center Utrecht, Utrecht University, Heidelberglaan 100, 3584 CX Utrecht, The Netherlands

**Keywords:** Optoacoustic technologies, Breast neoplasms, Molecular imaging

## Abstract

**Aim:**

This study was conducted in order to investigate the role of gray-scale ultrasound (US) and optoacoustic imaging combined with gray-scale ultrasound (OA/US) to better differentiate between breast cancer molecular subtypes.

**Materials and methods:**

All 67 malignant masses included in the Maestro trial were retrospectively reviewed to compare US and OA/US feature scores and histopathological findings. Kruskal–Wallis tests were used to analyze the relationship between US and OA/US features and molecular subtypes of breast cancer. If a significant relationship was found, additional Wilcoxon–Mann–Whitney tests were used to identify the differences between molecular subtype groups.

**Results:**

US sound transmission helped to differentiate between LUMA and LUMB, LUMB and TNBC, and LUMB and all other molecular subtypes combined (*p* values < 0.05). Regarding OA/US features, the sum of internal features helped to differentiate between TNBC and HER2-enriched subtypes (*p* = 0.049). Internal vessels (*p* = 0.025), sum of all internal features (*p* = 0.019), and sum of internal and external features (*p* = 0.028) helped to differentiate between LUMA and LUMB. All internal features, the sum of all internal features, the sum of all internal and external features, and the ratio of internal and external features helped to differentiate between LUMA and TNBC. The same features also helped to differentiate between LUMA and TNBC from other molecular subtypes (*p* values < 0.05).

**Conclusions:**

The use of OA/US might help radiologists to better differentiate between breast cancer molecular subtypes. Further studies need to be carried out in order to validate these results.

**Key Points:**

*• The combination of functional and morphologic information provided by optoacoustic imaging (OA) combined with gray-scale US helped to differentiate between breast cancer molecular subtypes.*

**Electronic supplementary material:**

The online version of this article (10.1007/s00330-019-06262-0) contains supplementary material, which is available to authorized users.

## Introduction

Breast cancer is the most frequently occurring malignancy and most common cause of cancer-related death in women worldwide [1]. However, a combination of advances in breast cancer research, more effective treatments, introduction of screening programs, and improvement of diagnostic imaging tools has contributed to the increase of breast cancer survival rates in the last two decades [2–4].

Imaging plays a crucial role in detection, diagnosis, guiding biopsies and interventions, monitoring response to therapy, and surveillance of breast cancer [5]. Mammography, magnetic resonance imaging (MRI), and ultrasound (US) are the most important imaging modalities for evaluation of breast lesions. Recent studies have been directed toward developing and enhancing imaging methods to obtain functional information of breast tumors. This additional information may facilitate the recognition of breast cancer biomarkers, consequently facilitating clinical management and treatment planning [6, 7].

Angiogenesis has been recognized as one of the hallmarks of breast cancer. The production of new blood vessels is essential to support the development of malignant lesions once they become larger than 2 mm. Judah Folkman described the essential role of angiogenesis to provide nutrients and oxygen for tumor growth. He characterized malignant tumors as being basically “hot and bloody”, illustrating the typical flush perfusion and hyperemia found in these lesions [8–10]. Based on the principles of tumor neoangiogenesis and metabolism [11–16], the use of laser light to better characterize breast lesions is now being studied. Optoacoustics combined with gray-scale ultrasound (OA/US) is an imaging technique in which laser light is transmitted into the breast and its energy is absorbed primarily by blood. Red light (757 nm) is predominantly absorbed by deoxygenated blood, and near-infrared light (1064 nm) is predominantly absorbed by oxygenated blood. The absorbed dual wavelength laser light causes thermoelastic expansion of blood, which produces a pressure wave that is subsequently detected as an US wave by a piezoelectric US transducer. The optoacoustic (OA) signal is spatially co-registered and temporally interleaved in real time with gray-scale US, creating an oxygenation/deoxygenation blood map that gives both anatomic (US morphology and OA demonstration of angiogenesis) and functional information (relative oxygenation/ deoxygenation of hemoglobin). The fusion of anatomical and functional information provided by OA/US could help radiologists to better differentiate between benign and malignant lesions breast lesions. Butler et al [17] investigated the positive predictive value of optoacoustic ultrasound features, and the Pivotal [11] and Maestro [12] trials also evaluated the diagnostic utility of OA compared to US alone in differentiating benign from malignant breast masses. These studies concluded that OA/US might increase specificity in breast mass assessment, potentially reducing the number of false-positive examinations and biopsies of benign masses [11, 12, 17] Given these results, we hypothesize that OA/US might not only be useful to differentiate between benign and malignant masses, but it might also facilitate the differentiation between different subtypes of breast lesions. Studies have demonstrated that different molecular subtypes of breast cancer, such as luminal A (LUMA), luminal B (LUMB), HER2-enriched, and triple-negative breast cancers (TNBC) present distinct clinical behaviors, have different prognoses and require personalized treatment approaches [11, 18–20].

The goal of our study was to retrospectively determine the relationship between prospectively defined US and OA/US characteristics and histopathological prognostic indicators of breast masses, including the following: histologic grade, each of the three individual components of histologic grading (tubule formation, nuclear pleomorphism, and number of mitoses scores), and with secondary prognostic biomarkers such as continuous number of mitoses, HER2 receptor status, hormone receptor status, and Ki-67 proliferative index. We also assessed the relationships between US and OA/US feature scoring and molecular subtypes.

## Materials and methods

In this study, all malignant masses included in the Maestro [12] trial were retrospectively reviewed to compare US and OA/US feature scores and histopathological findings. The study took place in five centers in the Netherlands, and all OA/US images were prospectively acquired between March 2015 and February 2016. The ethical boards of the participating hospitals approved this study. Written informed consent was obtained from each participant. Women ≥ 18 years, with suspicious breast masses that were classified as BI-RADS 4a or 4b with conventional diagnostic US, were included in this study. Patients who were excluded from the study were those that (1) underwent previous biopsy or surgery of the mass of interest, (2) had previous biopsy or surgery within the same quadrant as the mass to be studied, (3) had mass of interest bigger than 3 cm, and (4) had more than three breast lesions. The full description of the inclusion and exclusion criteria as well as the primary objectives and other details of the Maestro trial design have been previously described [12].

We used a handheld US device that could perform both conventional gray-scale US alone and OA/US (fusion of US and laser light). The laser light is transmitted into the breast from the handheld duplex probe (OA and US) at two different wavelengths, which are used to image primarily oxygenated and deoxygenated hemoglobin (for more details, see Appendix Fig. 1). All masses that met the inclusion criteria were first evaluated with US and then reevaluated with OA/US. Five US and five OA/US feature scores were assigned for each mass (Appendix Tables 1 and 2 show the US and OA/US scoring system). This scoring system was developed from a previous trial (Pivotal [11]) and upon the current BI-RADS lexicon for gray-scale US [21]. Reference key images showing the minimum and maximum scores for each OA feature are displayed in Appendix Fig. 2. All OA/US examinations were performed by dedicated breast radiologists.

### Biopsy and treatment

OA/US scans were performed and interpreted, and results entered and locked in electronic case report forms prior to biopsy.

An independent central pathologist reviewed all biopsy and surgical specimens. Large-format sections (5 × 7 megacassettes) were obtained from surgical specimens to further facilitate the comparison between histologic characteristics and OA/US internal and external features in the external boundary and peripheral zones. The central pathology histopathologic diagnosis was the reference standard for OA/US comparison.

### Statistical methods

Given the non-normality of the distributions and small sample size, we chose nonparametric tests to analyze the data. Jonckheere–Terpstra tests were performed to evaluate if OA/US features helped differentiate between histologic grades of invasive carcinomas (I, II, III) and between each of the three components of histologic grading: tubule formation, nuclear pleomorphism, and number of mitoses scores. Spearman correlation was used to analyze the relationship between OA/US features and continuous number of mitoses index, percentages of estrogen receptor (ER) and progesterone receptor (PR) status, and continuous Ki-67 proliferative index. Kruskal–Wallis tests were used to analyze the relationship between OA/US feature scores and tumor margins (< 50%, > 50%, infiltrative and pushing) and the relationship between OA/US feature scores and HER2 status (0, 1+, 2+, 3+). The same statistical method was used to analyze the relationship between US and OA/US feature scores and molecular subtypes of breast cancer. If a significant relationship was found, additional Wilcoxon–Mann–Whitney tests were used to identify the differences between molecular subtype groups. Significance testing was performed for these supportive analyses without adjustment for multiple testing. Therefore, the *p* values reported in this study can be considered as descriptive statistics in this context.

Based on the St. Gallen International Expert Consensus of 2013 [22], breast tumors that were ER and PR positive and HER2 negative (IHC 0, 1+, or 2+ with nonamplified FISH) and had low levels of Ki-67 (< 20%) were classified as LUMA. Those that were ER positive and HER2 negative and had high levels of Ki-67 (≥ 20%) were considered as LUMB. Tumors that were ER positive and HER2 positive (IHC 3+ or 2+ FISH amplified) were also classified as LUMB, irrespective of PR or Ki-67 status. Tumors that were ER and PR negative and HER2 negative (IHC 0, 1+, or 2+ with nonamplified FISH) were classified as TNBC. Finally, ER and PR negative tumors that were HER2 positive (IHC 3+ or 2+ with FISH amplified) were classified as “HER2-enriched” cancers.

All statistical analysis was performed using SPSS, version 24.0 (IBM Corp).

## Results

Of the 215 biopsied masses enrolled in our study, histopathology was benign in 146, high risk in 2, and malignant in 67 (this last group was included in our analysis). The ages of patients with malignant lesions ranged from 30 to 84 (mean 57) years and ages of those with benign lesions ranged from 20 to 82 (mean 46) years. The mean maximum diameters were 1.43 for benign masses and 1.36 cm for malignant masses.

### Comparison between OA/US features and histopathological results

**Fig. 1:**
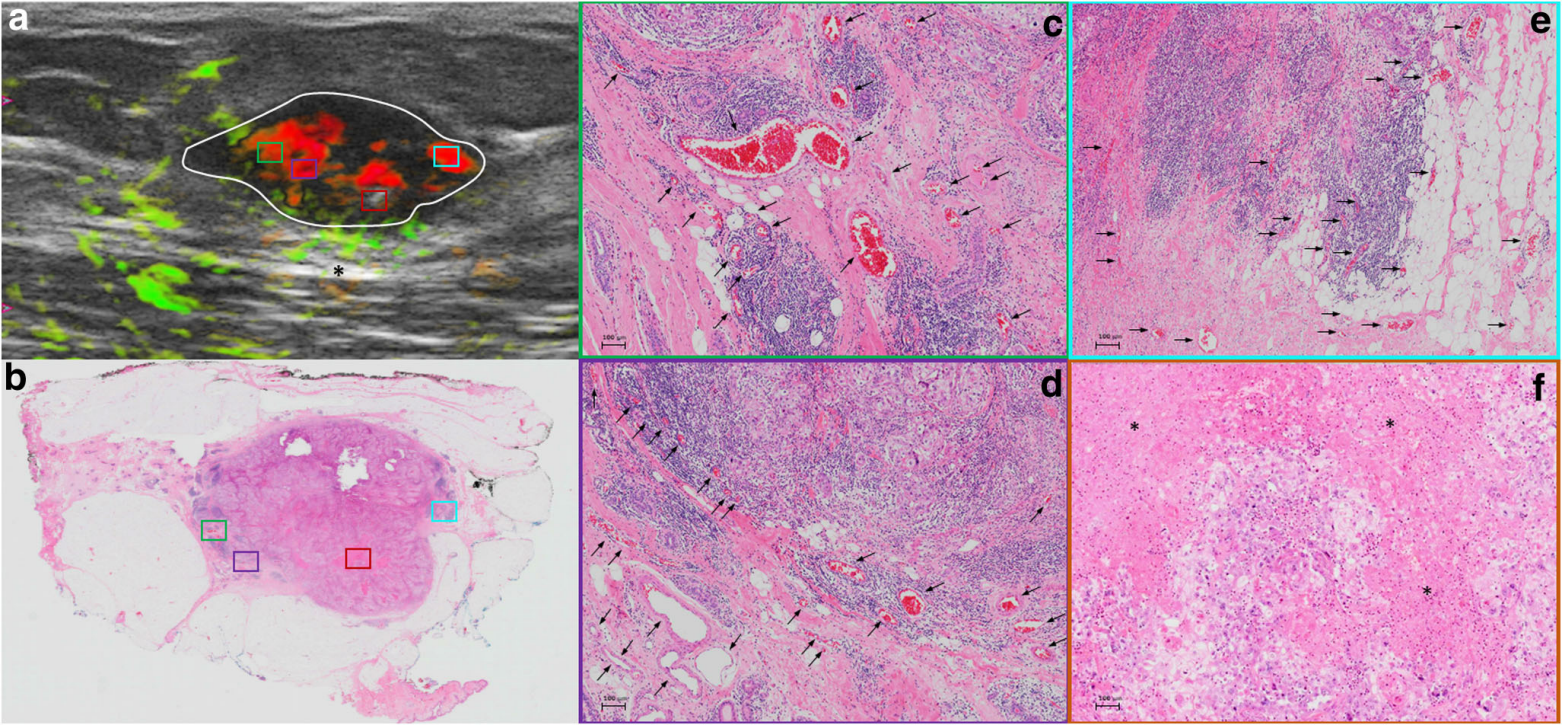
Triple-negative IDC, grade III, showing predominantly internal features at OA/US. A comparison between OA/US image (**a**) and the 5 × 7 megacassette surgical specimen (**b**) can be seen. The colored rectangles (green, orange, purple, and aqua color) seen on OA/US (**a**) and surgical specimen (**b**) are magnified in **c**–**f** (the correspondent magnified areas can be seen according to the color of the frame surrounding **c**–**f**). The internal vessels are seen as red blush areas in the OA/US map and correspond to the vessels seen on **c**–**e** (black arrows). Note that the slice thickness for OA/US is approximately 500–1000 μm, while the histopathological slide standard thickness is approximately 4–5 μm. Therefore, clusters of small vessels seen on the histopathological specimen are too small to be visible individually at OA/US. These tiny vessels can volume average and create an apparently larger single vessel on OA/US. **f** A completely avascular area of central necrosis within the mass seen both in OA/US (lack of signal) and histopathological specimen (asterisks). External OA/US findings are not seen, which is expected in TNBCs. Posterior enhancement can also be seen (black asterisk in **a**). TNBCs are usually more cellular and more water-rich tumors and often show enhancement through transmission

**Fig. 2:**
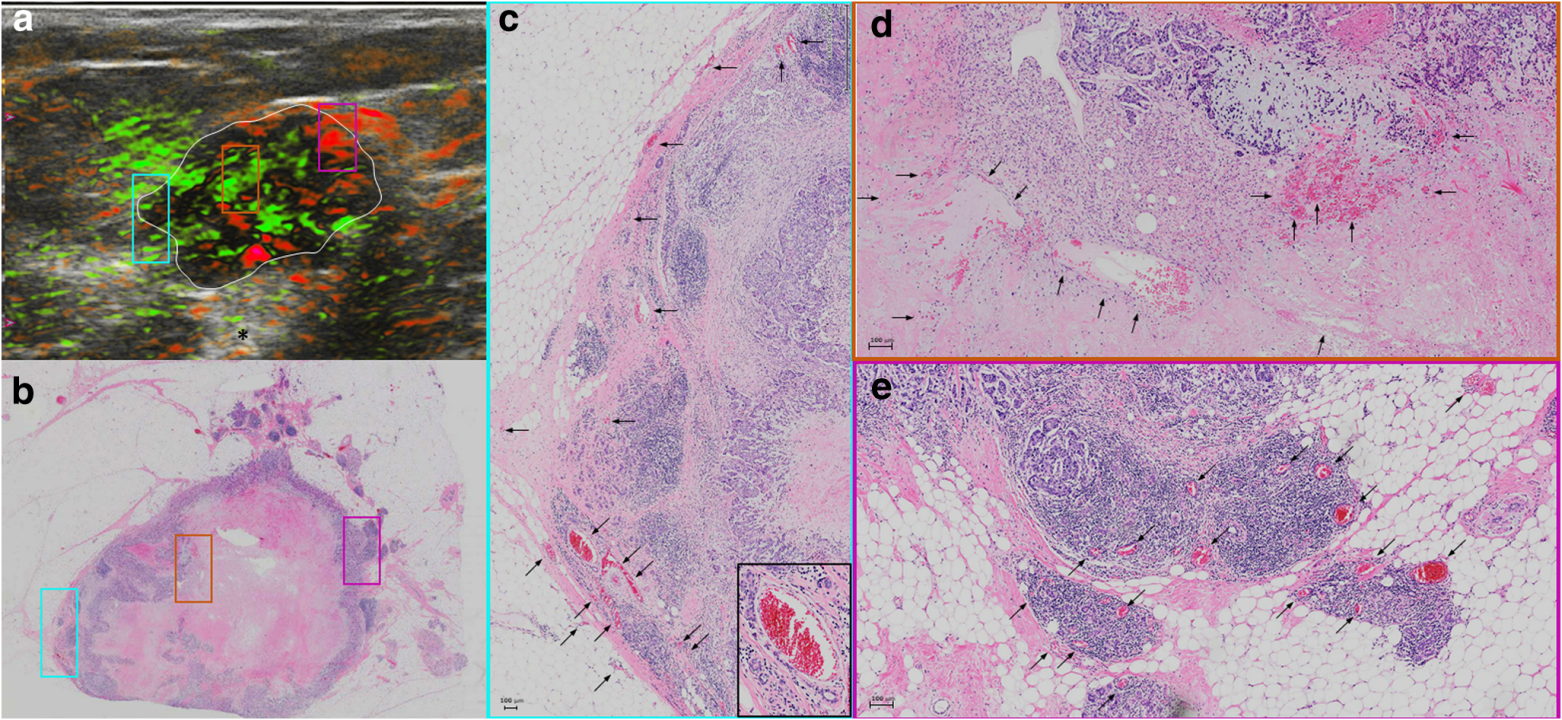
A triple-negative IDC, grade III, seen at OA/US (**a**) and at a histopathological megacassette (**b**). The correspondent areas highlighted with colored rectangles in **a** and **b** can be seen at higher magnification in **c**–**e**. The colored rims around **c**–**e** show which area corresponds to the magnification of the rectangles seen on **a** and **b**. This triple-negative mass shows predominantly internal vessels, as can be seen on **c**–**e** (black arrows). The inset at the lower right corner of **c** shows a vessel surrounded by lymphocytes. Lymphocytic infiltration is associated with a better prognosis in TNBCs. Areas rich in lymphocytes tend to be more vascular. It is unclear whether lymphocytes are arriving at these areas of the tumors because of the richly distributed leaky vessels, whether lymphocytes are contributing to formation of neovessels, or some combination of both. The leaky vessels also contribute to the higher presence of water in these tumors, resulting in posterior enhancement (black asterisk in **a**)

**Fig. 3:**
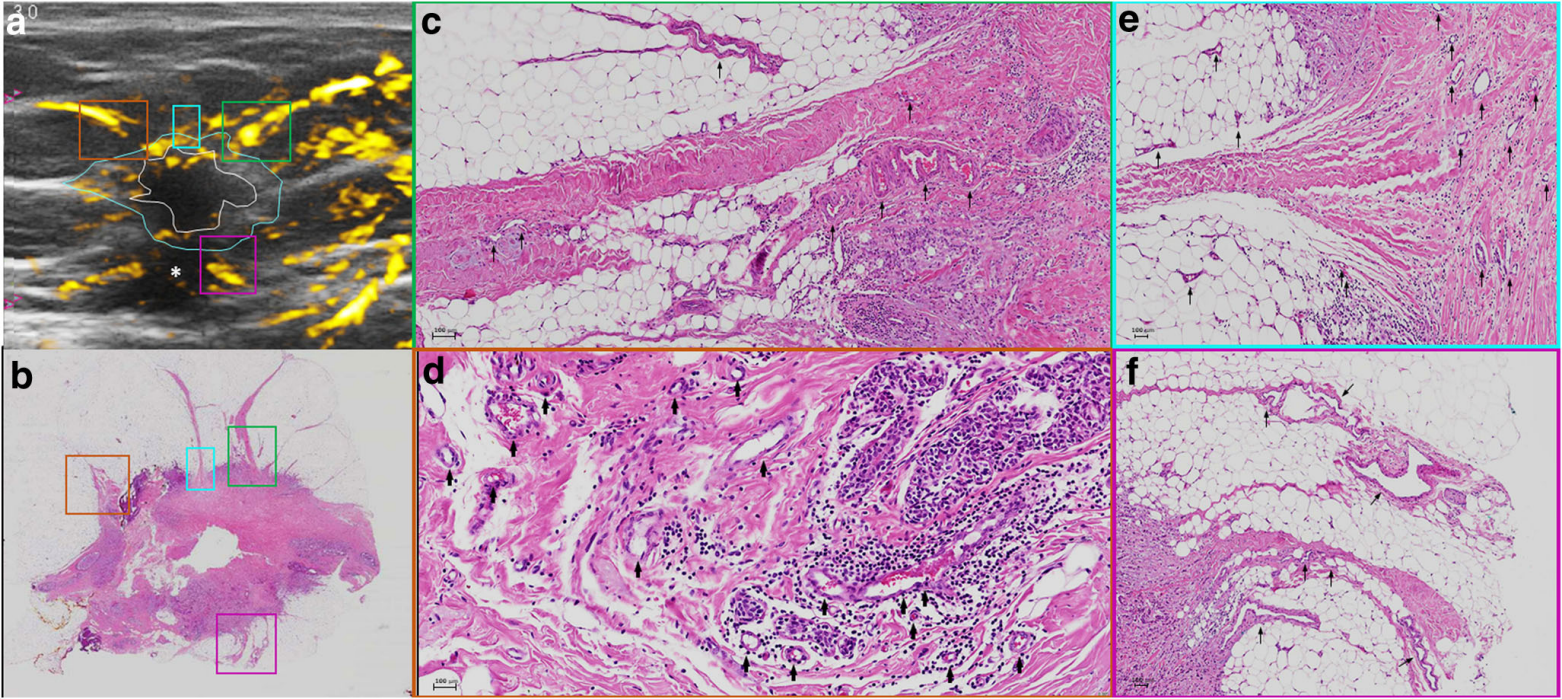
An example of a LUMA IDC, grade II. **a** The central nidus of the lesion (white ROI) and the boundary zone of the same lesion (aqua color ROI) on the total hemoglobin map (oxygenated and deoxygenated hemoglobin added together). The total hemoglobin map tends to be the best in showing peripheral radiating vessels in OA/US. **b** The megacassette surgical specimen. Notice the remarkable difference between LUMAs and TNBC: while TNBC are usually more well-circumscribed (round) and have mostly internal findings at OA/US, LUMAs usually show abundant external peripheral zone radiating vessels (**a**) and plentiful spicules and/or retracted Cooper’s ligaments around the mass (**b**), but a relative paucity of internal OA/US findings (central nidus in **a**). The radiating vessels (external OA/US findings) were highlighted with colored rectangles in **a** and **b** and magnified in **c**–**f** (black arrows show the vessel distribution). White asterisk in **a** shows posterior acoustic shadowing. LUMAs are usually relatively hypocellular and are largely comprised of fibrosis and desmoplasia, are relatively water-poor, and give rise to posterior acoustic shadowing

**Fig. 4:**
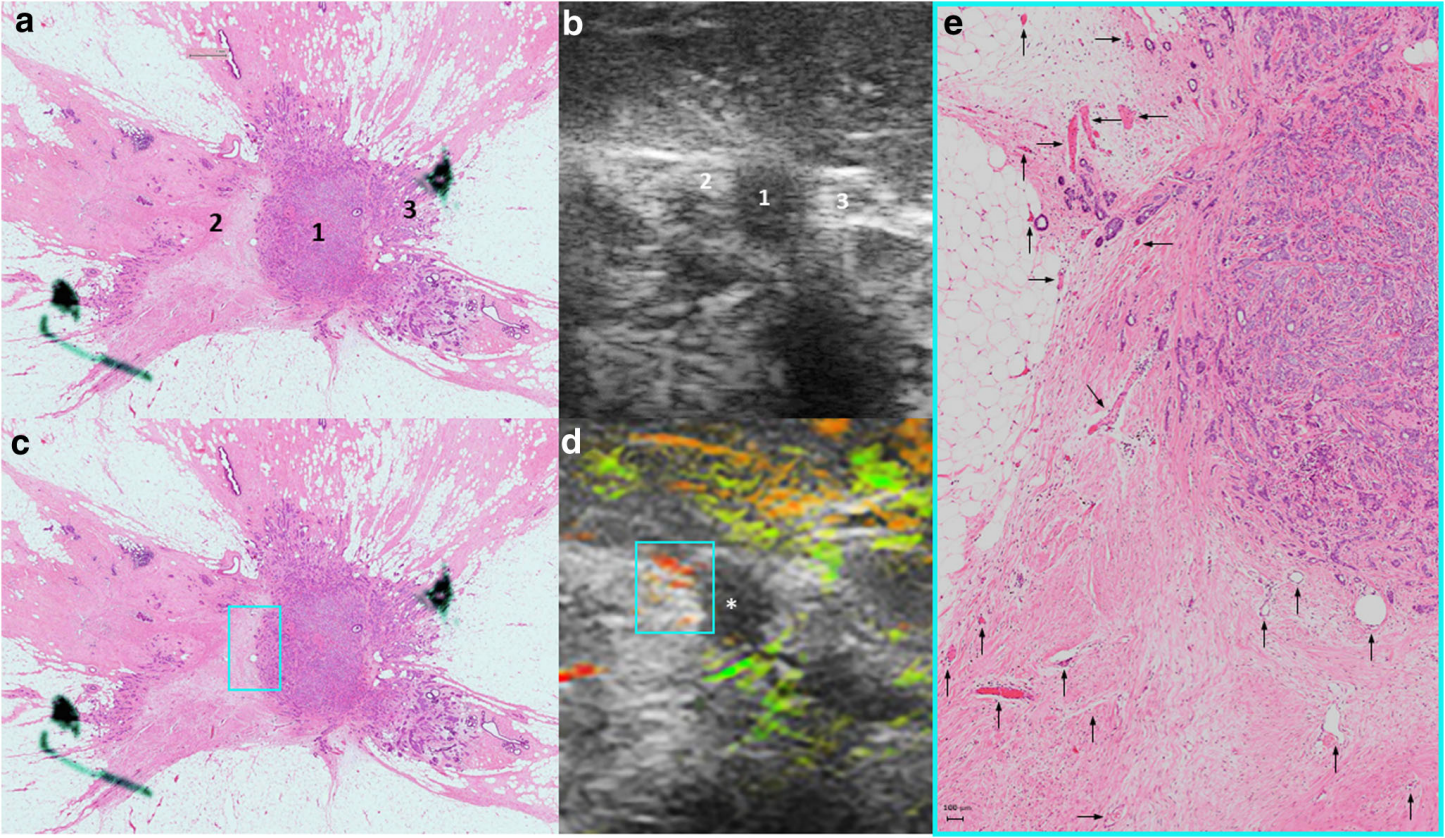
LUMA IDC, grade I, showing important boundary/peripheral zone spiculations seen both in the histopathological specimen (numbers 2 and 3 in **a**) and in the gray-scale US images (numbers 2 and 3 in **b**). Number 1 in **a** and **b** represents the central nidus of the mass. Radiating vessels seen on the left side of the lesion (aqua color squares seen in **c** and **d**) are magnified and highlighted with black arrows in **e**. OA/US shows a paucity of internal findings (white asterisk in **d**)

**Fig. 5:**
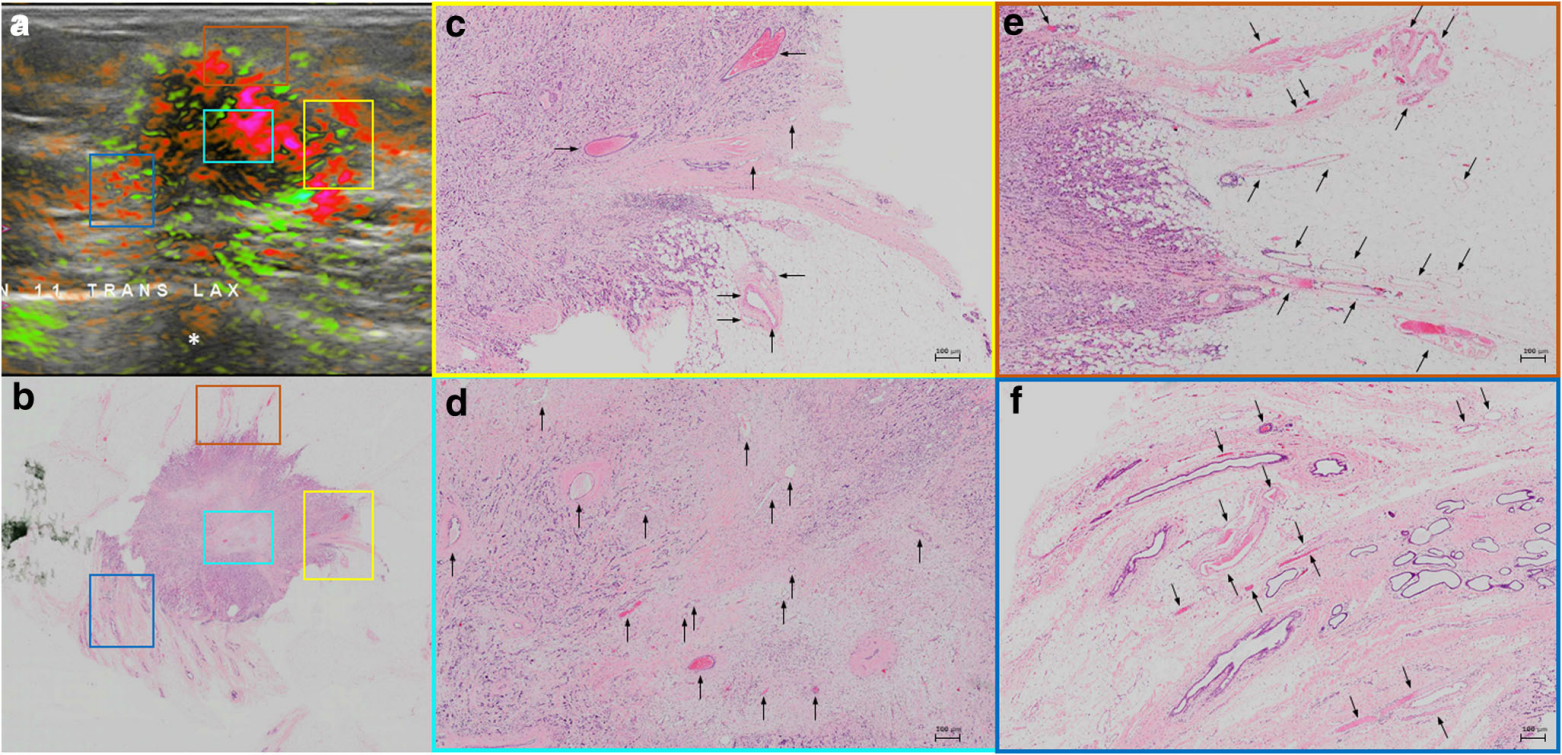
An example of LUMB (IDC, grade II)**.** These tumors are characterized by abundant internal and external findings simultaneously. **a** Important internal and external blush (aqua color, orange and yellow squares), as well as peripheral radiating vessels (blue rectangles). The correspondent areas in the pathological specimen can be seen in **b** (colored rectangles). Spiculations are seen around the mass (**b**). Black arrows highlight the vessels. Note that in **e**, short boundary zone neovessels are oriented roughly perpendicular to the surface of the internal zone, boundary zone “whiskers.” In the OA/US boundary zone, neovessels in grade I and II tumors typically orient roughly perpendicular to the surface of the tumor, while grade III invasive cancers tend to have dilated tortuous vessel that are not perpendicularly oriented. In grades I or II invasive breast cancers, boundary zone neovessels apparently use perpendicularly oriented TAC3 collagen fibers as infrastructure on which to form, accounting for their perpendicular orientation. Note also that in **f** that the vessels are interspersed between ductal structures (with purple duct epithelium). LUMB cancers are usually more water-rich than LUMA carcinomas. White asterisk in **a** shows partial acoustic shadowing, but not as prominent as the acoustic shadowing observed in Fig. 3a. LUMB cancers tend to have peripheral radiating vessels similar to those in LUMA cancers but tend to have internal vascularity more similar to that of TNBCs. Thus, LUMB cancers have an appearance that lies between those of LUMA and TNBC subgroup cancers. LUMB cancers are more often positive in all three zones and tend to have higher OA/US feature scores when compared to LUMA tumors

**Fig. 6:**
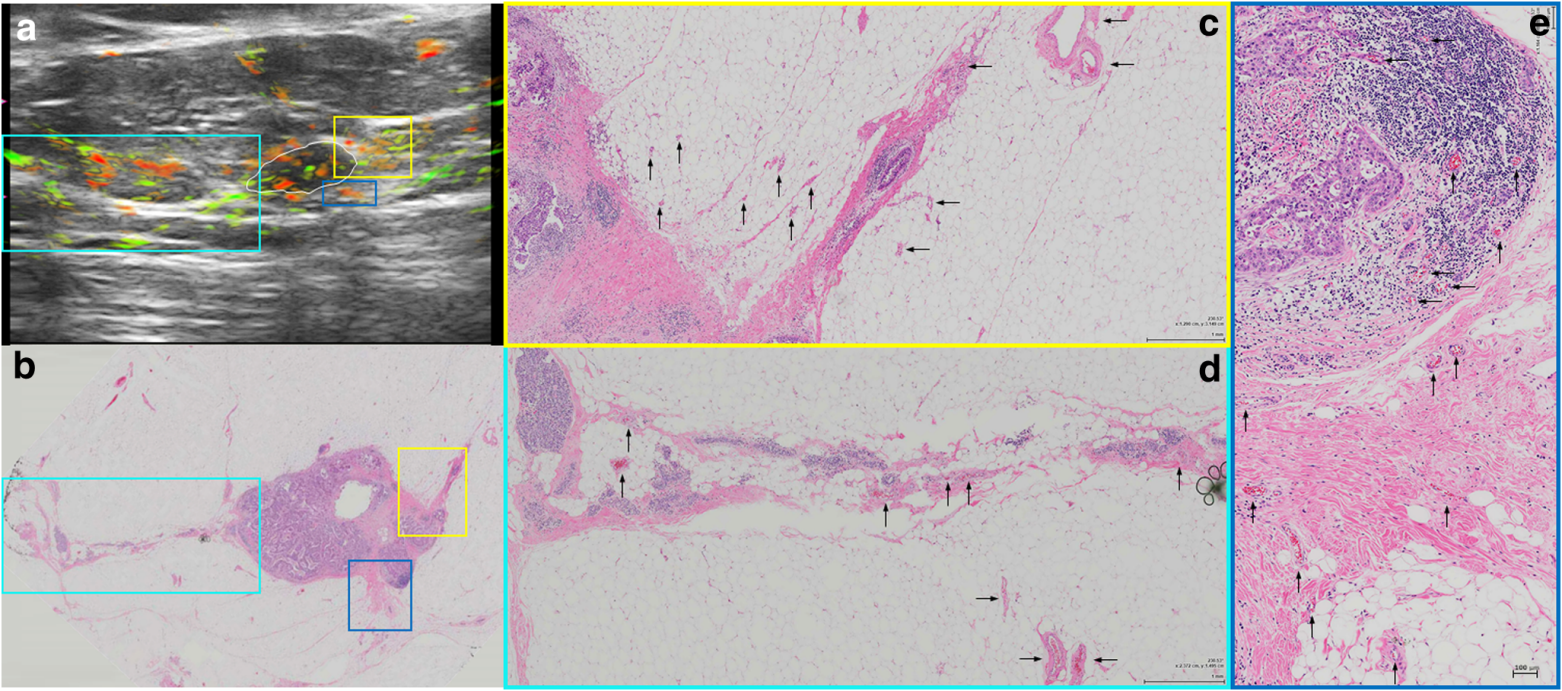
A HER2-enriched IDC, grade III, showing important peripheral findings at OA/US (aqua color, yellow and blue squares in **a**). The radiating vessels course within or parallel to and beside spiculations (aqua color square in **b**) and/or Cooper’s ligaments (yellow square in **b**). The vessels present in the areas of the colored rectangles seen in **a** and **b** are magnified and highlighted with black arrows in **c**–**e**). Notice that, according to our findings, HER2-enriched tumors present in a similar way as LUMAs, with important external/peripheral findings and poor internal findings (as seen in this case)

Table 1 shows the primary histopathologic diagnoses for benign and malignant masses found in our study. Tables 2 and 3 show the *p* values, medians, the 25th and 75th percentiles, and the interquartile ranges (IQRs) for the comparisons between OA/US feature scores and histopathological results of invasive breast carcinomas, including tubule formation, nuclear pleomorphism, number of mitoses scores, tumor margins, HER2 receptor status, ER and PR status, and continuous Ki-67 index. Table 4 shows the *p* values obtained by the Kruskal–Wallis tests when comparing the performance of US vs OA/US in accessing breast cancer molecular subtypes. Table 5 shows the *p* values, medians, the 25th and 75th percentiles, and the IQRs for the pairwise comparisons between US and OA/US feature scores and breast cancer molecular subtypes. The pairwise comparisons were only obtained for features that were found to be significant by the Kruskal–Wallis tests (Table 4). Figures 1, 2, 3, 4, 5, and 6 illustrate some of the differences in US and OA/US features between LUMA, TNBC, LUMB, and HER2-enriched breast cancers.

**Table 1:** Primary histology type of benign and malignant masses

	Frequency
Primary histology benign masses	
Benign phyllodes tumor	3
Fat necrosis	1
Fibroadenoma	75
Other	61
Papilloma	6
Total	146
Primary histology malignant masses	
DCIS	2
Invasive breast cancer	59
Lymphoma	1
Other	5
Total	67
Primary histology high-risk masses	
Total	2
Primary histology all masses	
Total	215

**Table 2:** *p* values, medians, 25th and 75th percentiles, and IQRs regarding the comparisons between OA/US feature scores and histopathological results: tubule formation, nuclear pleomorphism and number of mitoses scores, continuous total number of mitoses, and invasive cancer grades

	Tubule formation scores^a, b^ (*N* = 59)	Nuclear pleomorphism scores^a, b^ (*N* = 59)	Number of mitoses scores^a, b^ (*N* = 59)	Tumor margin scores^c, d^ (*N* = 47)	HER2 receptor status (0, 1+, 2+, 3+)^c, d^ (*N* = 64)	Invasive cancer histologic grades (I, II, and III) (*N* = 59)^a, b^
OA/US sum of 3 internal feature scores	*p* = 0.537 Median (25th, 75th) = 6.0 (4.0, 8.0) IQR = 4.0	*p* = 0.118 Median (25th, 75th) = 6.0 (4.0, 8.0) IQR = 4.0	*p* = 0.053 Median (25th, 75th) = 6.0 (4.0, 8.0) IQR = 4.0	*p* = 0.166 Median (25th, 75th) = 6.0 (4.0, 9.0) IQR = 5.0	*p* = 0.697 Median (25th, 75th) = 7.0 (4.0, 8.0) IQR = 4.0	*p* = 0.099 Median (25th, 75th) = 6.0 (4.0, 8.0) IQR = 4.0
OA/US sum of 2 external feature scores	*p* = 0.144 Median (25th, 75th) = 7.0 (3.0, 9.0) IQR = 6.0	*p* = 0.217 Median (25th, 75th) = 7.0 (3.0, 9.0) IQR = 6.0	*p* = 0.380 Median (25th, 75th) = 7.0 (3.0, 9.0) IQR = 6.0	*p* = 0.382 Median (25th, 75th) = 6.0 (4.0, 9.0) IQR = 5.0	*p* = 0.637 Median (25th, 75th) = 7.0 (4.0, 9.0) IQR = 5.0	*p* = 0.129 Median (25th, 75th) = 7.0 (3.0, 9.0) IQR = 6.0
OA/US sum of all 5 feature scores	*p* = 0.135 Median (25th, 75th) = 13.0 (9.0, 16.0) IQR = 7.0	*p* = 0.130 Median (25th, 75th) = 13.0 (9.0, 16.0) IQR = 7.0	*p* = 0.143 Median (25th, 75th) = 13.0 (9.0, 16.0) IQR = 7.0	*p* = 0.336 Median (25th, 75th) = 13.0 (9.0, 17.0) IQR = 8.0	*p* = 0.943 Median (25th, 75th) = 13.0 (9.0, 16.0) IQR = 7.0	*p* = 0.068 Median (25th, 75th) = 13.0 (9.0, 16.0) IQR = 7.0

^a^*p* values generated by the Jonckheere–Terpstra test

^b^Medians, percentiles, and IQRs only calculated for invasive carcinomas

^c^*p* values generated by the Kruskal–Wallis test

^d^Excluding nonrecorded cases

**Table 3:** *p* values, medians, 25th and 75th percentiles, and IQRs regarding the correlations between OA/US feature scores and secondary histopathological indicators: continuous number of mitoses index, ER status PR status, and continuous Ki-67 index

	Continuous number of mitoses index^a, b^ (*N* = 59)	ER%^a^ (*N* = 67)	PR%^a^ (*N* = 67)	Ki67%^a, c^ (*N* = 60)
OA/US sum of 3 internal feature scores	*p* = 0.035 Median (25th, 75th) = 6.0 (4.0, 8.0) IQR = 4.0	*p* = 0.033 Median (25th, 75th) = 7.0 (4.0, 8.0) IQR = 4.0	*p* = 0.333 Median (25th, 75th) = 7.0 (4.0, 8.0) IQR = 4.0	*p* = 0.009 Median (25th, 75th) = 6.0 (4.0, 8.0) IQR = 4.0 9
OA/US sum of 2 external feature scores	*p* = 0.296 Median (25th, 75th) = 7.0 (3.0, 9.0) IQR = 6.0	*p* = 0.878 Median (25th, 75th) = 7.0 (4.0, 9.0) IQR = 5.0	*p* = 0.830 Median (25th, 75th) = 7.0 (4.0, 9.0) IQR = 5.0	*p* = 0.894 Median (25th, 75th) = 7.0 (4.0, 9.0) IQR = 5.0
OA/US sum of all 5 feature scores	*p* = 0.107 Median (25th, 75th) = 13.0 (9.0, 16.0) IQR = 7.0	*p* = 0.124 Median (25th, 75th) = 13.0 (9.0, 17.0) IQR = 8.0	*p* = 0.400 Median (25th, 75th) = 13.0 (9.0, 17.0) IQR = 8.0	*p* = 0.157 Median (25th, 75th) = 14.0 (10.0, 17.0) IQR = 7.0

^a^*p* values generated by Spearman correlation

^b^Medians, percentiles, and IQRs only calculated for invasive carcinomas

^c^Excluding nonrecorded cases

**Table 4:** *p* values returned by the Kruskal–Wallis tests when comparing the performance of US vs OA/US in accessing breast cancer molecular subtypes: LUMA, LUMB, TNBC, and HER2-enriched breast cancers (*N* = 59)

	US shape	US internal echotexture	US sound transmission (posterior features)	US boundary zone	US peripheral zone	US sum of 3 internal feature scores	US sum of 2 external feature scores	US ratio of sum of 3 internal/sum of 2 external feature scores	US sum of all 5 feature scores
*p* values Kruskal–Wallis test	0.720	0.198	0.041*	0.053	0.219	0.301	0.203	0.335	0.240
	OA/US internal vessels scores	OA/US internal deoxygenated blush scores	OA/US internal hemoglobin scores	OA/US boundary zone scores	OA/US peripheral zone scores	OA/US sum of 3 internal feature scores	OA/US sum of 2 external feature scores	OA/US ratio of sum of total internal/sum of total external features	OA/US sum of total internal and external features
*p* values Kruskal–Wallis test	0.006*	0.024*	0.034*	0.487	0.191	0.003*	0.326	0.039*	0.028*

*p values < 0.05

**Table 5:** *p* values, medians, 25th and 75th percentiles, and IQRs for the pairwise comparisons between OA/US features and breast cancer molecular subtypes: LUMA, LUMB, TNBC, and HER2-enriched (HER2-E) breast cancers (*N* = 59)

		LUMA	LUMB	TNBC	HER2-E	LUMA vs LUMB	LUMA vs TNBC	LUMA vs HER2-E	LUMB vs TNBC	LUMB vs HER2-E	TNBC vs HER2-E	LUMA vs others	LUMB vs others	TNBC vs others	HER2-E vs others	
		(*n* = 25)	(*n* = 15)	(*n* = 15)	(*n* = 4)											
US sound transmission						*p* = 0.028	*p* = 0.312	*p* = 0.787	*p* = 0.006	*p* = 0.274	*p* = 0.716	*p* = 0.551	*p* = 0.006	*p* = 0.067	*p* = 0.731	
	Median (25th, 75th)	1.00 (1.00, 3.00)	2.00 (2.00, 4.00)	1.00 (1.00, 2.00)	2.00 (0.25, 3.00)											
	IQR	2.00	2.00	1.00	− 2.75											
OA/US internal vessel scores						*p* = 0.025	*p* = 0.003	*p* = 1.000	*p* = 0.276	*p* = 0.068	*p* = 0.062	*p* = 0.003	*p* = 0.226	*p* = 0.007	*p* = 0.291	
	Median (25th, 75th)	2.0 (1.0, 2.0)	2.0 (2.0, 3.0)	3.0 (2.0, 5.0)	2.0 (1.2, 2.0)											
	IQR	1.00	1.00	3.00	0.8											
OA/US internal deoxygenated blush scores						*p* = 0.090	*p* = 0.004	*p* = 0.713	*p* = 0.215	*p* = 0.370	*p* = 0.104	*p* = 0.010	*p* = 0.498	*p* = 0.009	*p* = 0.563	
	Median (25th, 75th)	1.0 (1.0, 2.5)	2.0 (1.0, 3.0)	3.0 (2.0, 4.0)	2.0 (1.2, 2.0)											
	IQR	1.5	2.00	2.00	0.8											
OA/US internal hemoglobin scores						*p* = 0.068	*p* = 0.005	*p* = 0.229	*p* = 0.429	*p* = 0.835	*p* = 0.327	*p* = 0.005	*p* = 0.422	*p* = 0.025	*p* = 0.849	
	Median (25th, 75th)	1.0 (1.0, 2.0)	2.0 (1.0, 4.0)	3.0 (2.0, 4.0)	2.0 (1.2, 2.7)											
	IQR	1.0	3.0	2.0	1.5											
OA/US sum of 3 internal feature scores						*p* = 0.019	*p* = 0.001	*p* = 0.523	*p* = 0.202	*p* = 0.265	*p* = 0.049	*p* = 0.001	*p* = 0.265	*p* = 0.003	*p* = 0.533	
	Median (25th, 75th)	4.0 (3.0, 7.0)	7.0 (5.0, 9.0)	8.0 (6.0, 12.0)	5.5 (4.2, 6.8)											
	IQR	4.0	4.0	6.0	2.6											
OA/US ratio sum of 3 internal/sum of 2 external feature scores						*p* = 0.164	*p* = 0.006	*p* = 0.373	*p* = 0.074	*p* = 0.961	*p* = 0.530	*p* = 0.013	*p* = 0.909	*p* = 0.011	*p* = 0.808	
	Median (25th, 75th)	1.0 (0.4, 1.8)	1.0 (0.7, 1.3)	1.4 (0.8, 2.5)	0.9 (0.9, 1.7)											
	IQR	1.4	0.6	1.7	0.8											
OA/US sum of all 5 internal and external feature scores						*p* = 0.028	*p* = 0.010	*p* = 0.898	*p* = 0.770	*p* = 0.175	*p* = 0.130	*p* = 0.008	*p* = 0.140	*p* = 0.048	*p* = 0.423	
	Median (25th, 75th)	11.0 (6.0, 14.5)	14.0 (12.0, 19.0)	15.0 (10.0, 18.0)	11.5 (7.0, 14.5)											
	IQR	8.5	7.0	8.0	7.5											

*p* values generated by the Wilcoxon–Mann–Whitney *U* tests. The pairwise comparisons were only obtained for features that were found to be significant by the Kruskal–Wallis tests (Table 4)

OA/US feature scores (internal, external, and total) did not help to distinguish between tubule formation, nuclear pleomorphism, number of mitoses scores, or histologic grades of invasive carcinomas (Table 2). HER2 receptor status and tumor borders also were not differentiated by OA/US features. Significant correlations were found between OA/US internal feature scores and continuous number of mitoses (*p* = 0.035), ER status (*p* = 0.033), and Ki-67 (*p* = 0.009) percentages (Table 3). Among US feature scores, sound transmission (Table 5) helped to differentiate between LUMA and LUMB (*p* = 0.028), as well as LUMB and TNBC (*p* = 0.006) and LUMB and all other molecular subtypes combined (*p* = 0.0069). Black and white asterisks in Figs. 1a, 2a, 3a, and 5a show sound transmission differences according to the molecular subtypes. Among OA/US feature scores (Table 5), internal vessel scores (*p* = 0.025), sum of all three internal feature scores (*p* = 0.019), and the sum of total internal and external feature scores (*p* = 0.028) helped to differentiate between LUMA and LUMB molecular subtypes. Figures 3 and 4 show the paucity of internal OA/US findings in LUMA carcinomas compared to the more exuberant internal findings of LUMB carcinomas, as seen in Fig. 5. Internal vessel scores (*p* = 0.003), internal blush scores (*p* = 0.004), total internal hemoglobin scores (*p* = 0.005), sum of three internal feature scores (*p* = 0.001), and the ratio between the sum of the three internal and the sum of the two external feature scores (*p* = 0.006), as well as the sum of all five internal and external feature scores (*p* = 0.010) helped to differentiate between LUMA and TNBC subtypes. Figures 1, 2, 3, and 4 show examples of typical OA/US and histopathological differences between LUMAs and TNBCs. TNBCs show rich internal findings at OA/US and relative lack of external peripheral zone findings (Figs. 1a, b and 2a, b), while LUMAs present with more conspicuous external peripheral zone radiating vessels (Fig. 3a), but reduced OA/US internal feature scores.

The sum of all three internal feature scores also helped to differentiate between TNBC and HER2-enriched subtypes (*p* = 0.049). Figure 6 shows an example of the relative paucity of internal findings in HER2-enriched carcinomas, similar to LUMA tumors. When comparing individual molecular subtypes with all other types combined, internal vessel score (*p* = 0.003), deoxygenated blush scores (*p* = 0.010), internal hemoglobin score (*p* = 0.005), sum of three internal feature scores (*p* = 0.001), the ratio of the sum of three internal and the sum of the two external feature scores (*p* = 0.013), and the sum of all five internal and external feature scores (*p* = 0.008) helped to differentiate between LUMAs and other molecular subtypes. The very same features also helped to differentiate between TNBCs and other molecular subtypes (Table 5).

## Discussion

One of our most interesting findings is the fact that US sound transmission feature helps to differentiate between LUMAs and LUMBs, as well as LUMBs and TNBCs and LUMBs and other molecular subtypes. The water content of a tumor has an impact in sound transmission and it is based upon three factors: cellularity, constituents of the extracellular matrix, and the host response to the tumor. LUMAs and low-grade invasive cancers are relatively hypocellular, have an extracellular matrix largely comprised of fibrosis, and incite a primarily desmoplastic host response. All three of these components are relatively water poor, tending to manifest with acoustic shadowing [23–26]. On the other hand, higher grade and more aggressive molecular subtypes, such as LUMBs and TNBCs, tend to be much more cellular, have extracellular matrices enriched in hydrophilic hyaluronic acid, and tend to incite a highly cellular lymphocytic response. Both tumor cells and lymphocytes contain more than 90% water [23–30]. The high tumor and host response cellularity together with hydrophilic extracellular matrix result in water-rich TNBCs that transmit sound better than normal breast tissue, manifesting with enhanced sound transmission [23–26]. This is especially true for grade III TNBCs [26–30].

Our results show that US sound transmission scores were effective. Nevertheless, the combination of functional and morphologic information provided by OA/US features was even more valuable to differentiate between breast cancer molecular subtypes, and the differences between molecular subtypes may justify these findings. LUMA tumors usually have low levels of proliferation-related genes, have mild/moderate cellularity, are usually of low histological grade, and have a better outcome when compared to LUMBs [23–25]. Compared to LUMAs, LUMB cancers are more often of higher histological grade and have higher proliferation rates, lower cellular cohesion, higher rates of necrosis, and a worse prognosis [23–25, 27, 28]. Notably, malignant stromal cells—mostly tumor-associated fibroblasts—which are more frequently found in LUMBs than in LUMAs—can induce tumor cell proliferation and also promote angiogenesis [30, 31]. This may explain the higher scores for internal vessel and summed three internal feature scores found in LUMB tumors in our study.

Compared to luminal subtypes, TNBC cancers are usually seen as round, oval, or lobulated masses [32–35]. Furthermore, TNBCs are classified as having high histologic grade, with central necrotic zones, cellular fibrous proliferation, pushing borders, perilobular and intratumoral lymphocytic inflammatory infiltration, and often having thick-walled vessels [36–38]. In our study, TNBC showed significantly higher medians (compared to LUMAs) for internal vessel, internal blush, internal hemoglobin, sum of three internal feature scores, and ratio between summed internal and sum of all five internal and external feature scores. Recent studies showed that B and T lymphocytes can exert protumor activity indirectly by regulating the activity of myeloid cells, including macrophages, mast cells, and monocytes [39–42]. In response to distinct signals, macrophages undergo polarization into two different states: M1 (classical) or M2 (alternative) [43], which is comparable to the differentiation of helper T cells into type 1 (Th1) or type 2 (Th2). M2 tumor-associated macrophages (TAMs) inhibit Th1 activity, promoting invasion, migration of tumor cells, and angiogenesis. Medrek et al prospectively analyzed 144 patients with invasive breast cancer and concluded that dense infiltration of tumor stroma by M2 macrophages positively correlates with TNBC and inversely correlates with LUMA breast cancers [44]. Therefore, the higher medians for OA/US features found in TNBC in our study are probably associated with the lymphocyte-facilitated angiogenesis and increased metabolic activity found within TNBC (compared to LUMA tumors) [23–30]. TNBCs that have higher percentages of tumor-infiltrating lymphocytes usually have a better response to chemotherapy and also show better prognosis than those with lower lymphocytic infiltration within the tumor stroma [45–47].

Many studies also showed that intense infiltration of tumor stroma by TAMs is significantly associated with high vascular endothelial growth factor (important controller of angiogenesis), higher blood microvessel density, and higher numbers of mitoses per 10 high power fields [48–50]. This may also explain the significant correlation between OA/US features and continuous number of mitoses found in our study.

Our results showed that OA/US feature scores assigned to LUMB subtypes were not significantly different than those assigned to TNBCs. Although LUMB tumors present lower cellularity and lower grade and less extensive necrosis than TNBCs, these differences are not as pronounced when comparing LUMAs vs TNBC [23–25].

Another interesting finding was the significant difference between TNBC and HER2-enriched regarding total internal features. These two types of tumors are known to have many overlapping characteristics: both of them are usually high grade, have low cell cohesion, and present with more extensive necrosis [25, 31, 51]. However, TNBCs usually have higher cellularity and tubular and syncytial cluster scores when compared to HER2-enriched breast cancers [25, 31, 51, 52], which may also explain the significantly higher total internal feature scores found in TNBCs. However, we had a small number of HER2 cases in our study and further research is necessary to confirm these results.

Our findings shed new light on the use of OA/US technology to help clinicians to better differentiate between breast cancer molecular subtypes. Molecular analysis requires specialized equipment and technical expertise, consequently increasing healthcare costs. Recent studies with small number of patients using MRI to better differentiate between breast cancer molecular subtypes presented reasonable results [53–55]. However, MRI is a costly (and not yet widely available) imaging technique. Breast tumors are usually heterogeneous, and biopsy may often be insufficient to assess intratumoral heterogeneity [56–58]. OA/US, on the other hand, might display the dominant feature of the whole tumor. OA/US features that suggest an aggressive tumor with a worse prognosis that is discordant with histopathologic biomarkers might indicate the need for more extensive histopathologic sampling. This does not necessarily indicate the need for rebiopsy or excision, but rather, a need for the pathologist to section and inspect more of the currently available specimen. Although it is unlikely that OA/US or any other imaging technique will make histologic biomarker analysis unnecessary, OA/US could still be useful as a prognostic biomarker.

The generalizability of these results is subject to certain limitations. First, the scope of this study was limited in terms of the number of patients, and the number of malignancies for each molecular subtype group was relatively low. Second, our statistical analysis was performed without adjustment for multiple testing, and future studies are needed to confirm the *p* values reported in this study.

Notwithstanding these limitations, the study suggests that both the functional and morphologic information provided by OA/US might help radiologists to better differentiate between breast cancer molecular subtypes. Nevertheless, this emerging technique is in its infancy and more studies with larger sample sizes are needed to confirm these preliminary results.

## Electronic supplementary material

ESM 1 (DOCX 906 kb)

## Abbreviations

BI-RADS: Breast Imaging-Reporting and Data System
ER: Estrogen receptor
HER2: Human epidermal growth factor receptor-type 2
HER2-E: Human epidermal growth factor receptor-type 2—enriched
IDC: Invasive ductal carcinoma
IHC: Immunohistochemistry
IQRs: Interquartile ranges
LUMA: Luminal A
LUMB: Luminal B
MRI: Magnetic resonance imaging
OA: Optoacoustic
OA/US: Optoacoustic imaging combined with gray-scale ultrasound
PR: Progesterone-receptor
TAMs: Tumor-associated macrophages
Th1: T-cell type 1
Th2: T-cell type 2
TNBC: Triple-negative breast cancer
US: Ultrasound
