# Supplementary material for: Optoacoustic imaging of the breast: correlation with histopathology and histopathologic biomarkers
Source: Eur Radiol. 2019 May 27;29(12):6728–40. doi: 10.1007/s00330-019-06262-0 (PMC6828639; doi:10.1007/s00330-019-06262-0)

Supplementary Material

**Table 1 (Appendix). US (gray-scale US) Internal and External Feature Score**

| Internal Features | US Shape Scores | |
| --- | --- | --- |
|  | 0 | Ovoid, parallel orientation, (wider than tall), >2/1 ratio max width to AP dimension |
|  | 1 | Ovoid, parallel orientation, (wider than tall), < 2/1 ratio width to AP |
|  | 2 | Round |
|  | 3 | Irregular without angles, parallel in orientation |
|  | 4 | Irregular without angles, non-parallel orientation, (taller than wide), |
|  | 5 | Irregular with angles, parallel or non-parallel orientation |
|  | **US Internal Texture Scores** | |
|  | 0 | Homogeneous hyperehoic |
|  | 1 | Complex mixed cystic and solid |
|  | 2 | Homogeneous isoechoic or mildly hypoechoic |
|  | 3 | Heterogeneous |
|  | 4 | Heterogeneous with internal microcalcifications |
|  | 5 | Homogeneous severely hypoechoic |
|  | **US Sound Transmission Scores** | |
|  | 0 | Enhanced |
|  | 1 | Normal |
|  | 2 | Mixed normal and enhanced |
|  | 3 | Mixed enhanced and partial shadowing |
|  | 4 | Partial shadowing and/or weak shadowing |
|  | 5 | Complete and strong shadowing (obscures posterior margin of mass) |
| External Features | **US Boundary Zone Scores** | |
|  | 0 | Well-circumscribed with complete thin hyperechoic capsule |
|  | 1 | Well-circumscribed with partial thin hyperechoic capsule |
|  | 2 | Thick well-defined capsule |
|  | 3 | Well-circumscribed, but without thin hyperechoic capsule |
|  | 4 | Indistinct margin |
|  | 5 | Thick ill-defined echogenic rim. (halo) in boundary zone |
|  | 6 | Frank hypoechoic and/or hyperechoic short spiculations within boundary zone |
|  | **US Peripheral Zone Scores** | |
|  | 0 | Normal tissue |
|  | 1 | Critical angle shadowing in surrounding tissues |
|  | 2 | Surrounding ducts enlarged without microcalcifications (duct extension or branch pattern) |
|  | 3 | Surrounding enlarged ducts containing microcalcifications |
|  | 4 | Peripheral long hyperechoic spicules or interrupted tissue planes |
|  | 5 | Spicules and thickened coopers’ ligaments/retracted or thick skin |

**Table 2 (Appendix). OA/US (Optoacoustics combined with gray-scale US) Internal and External Feature Scores**

| Internal Features | OA/US Internal Vascularity and Deoxygenation (Vessel Scores) | |
| --- | --- | --- |
|  | 0 | No internal vessels |
|  | 1 | Normal internal vessels without branches, red or green |
|  | 2 | Normal internal vessels with branches, mostly green |
|  | 3 | Internal speckle; green = red in amount and less red than background |
|  | 4 | Internal speckle or signal; red > green and red > background |
|  | 5 | Multiple internal red vessels |
|  | **OA/US Internal Tumor Blush and Deoxygenation (Blush Scores)** | |
|  | 0 | No internal vessels |
|  | 1 | Minimal internal speckle, all green |
|  | 2 | Mild internal speckle; red=green and red + green < background |
|  | 3 | Mild internal speckle; red > green and both < background |
|  | 4 | Moderate internal speckle; red > green and red also > background |
|  | 5 | Red blush almost fills lesion |
|  | **OA/US Relative Internal Hemoglobin (Hemoglobin [Hgb] Scores)** | |
|  | 0 | No internal hemoglobin (Hgb) |
|  | 1 | Minimal internal Hgb, less Hgb than background |
|  | 2 | Minimal internal Hgb in discrete vessels, Hgb = background |
|  | 3 | Moderate internal Hgb in discrete vessels, Hgb = background |
|  | 4 | Many large internal vessels containing Hgb amount > background |
|  | 5 | Many large Hgb filled vessels almost fill central nidus of mass |
| External Features | **OA/US External Boundary Zone (BZ) Vascularity and Deoxygenation (BZ Scores)** | |
|  | 0 | No capsular/BZ vessels |
|  | 1 | Normal capsular/ BZ vessel(s) without branches (long, curved, parallel to capsule, not perpendicular to capsule) |
|  | 2 | Normal capsular/ BZ vessel(s) with normal tapering acutely angled branches, mostly green |
|  | 3 | Capsular/ BZ speckle; green = red; red < background red |
|  | 4 | Capsular/ BZ speckle; red > green; red > background red |
|  | 5 | ≥3 capsular/ BZ red vessels, some perpendicular |
|  | 6 | Boundary zone deoxygenated blush (complete or partial) |
|  | **OA/US Peripheral Zone Radiating Vessels Score (Peripheral Zone Scores)** | |
|  | 0 | No peripheral zone peri-tumoral vessels |
|  | 1 | Normal non-branching or branching peripheral zone feeding or draining vessels, at least one green, not in a radiating pattern |
|  | 2 | Cluster of enlarged tortuous vessels in the peripheral zone near the mass, non-radiating |
|  | 3 | 1 or 2 peripheral zone radiating vessels |
|  | 4 | > 2 peripheral zone radiating vessels on one side of the mass |
|  | 5 | > 2 peripheral zone radiating vessels on more than one side of the mass |

**Appendix Figure 1**


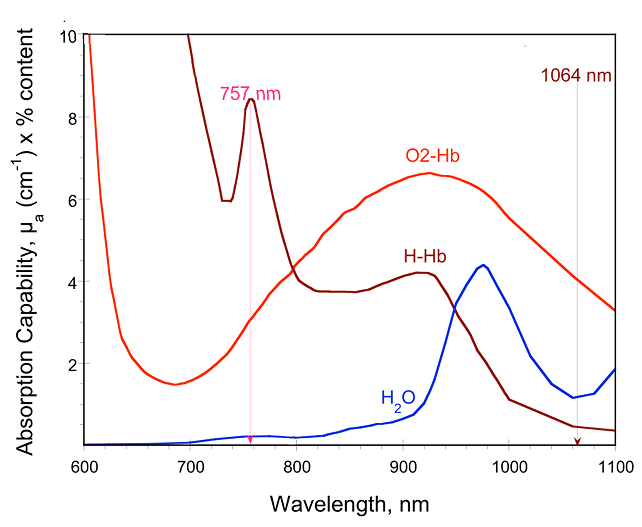


**Appendix Figure 2**


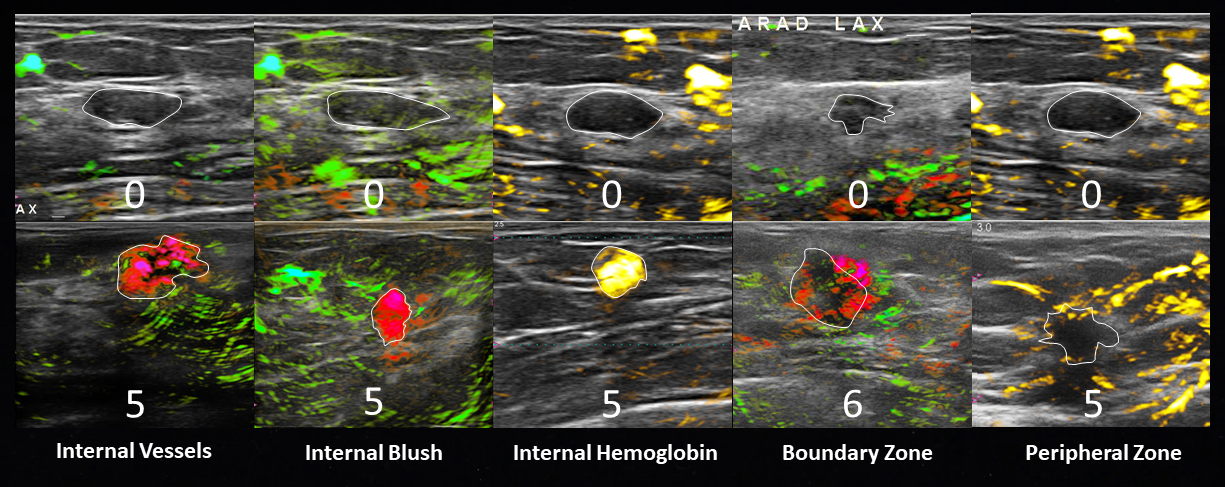

Supplement: Supplementary file 1 — (DOCX 906 kb) [file 330_2019_6262_MOESM1_ESM.docx]
